# Domestic Summary

**Published:** 1838-11-21

**Authors:** 


### DOMESTIC SUMMARY.

*Dr. Glizer on Dysentery.*—Some interesting remarks on this subject have been communicated to us by Dr. E. W. Glizer, of Mercer, Pa. The press of clinical matter, which is just now accumulating upon our hands, obliges us to condense this paper, from which we, however, extract some valuable information. "Dysentery," Dr. G. says, "is a very common epidemic in Mercer county; in the months of August and September, it is emphatically the terror of this section. During eight years that I have been in practice here, no one has passed without the prevalence of this affection to a greater or less extent. In 1834 it was unusually rife. My practice was founded on pathological views similar to those which I hold in reference to cholera morbus, diarrhœa, &c., which always precede dysentery in this region, viz., a cessation of the functions of the liver and skin, and consequent derangement of the digestive apparatus. I gave alternate doses of calomel and ipecacuanha, followed by castor oil, with diluted drinks, and sinapisms over the abdomen. With this practice I had had satisfactory success in the cholera infantum and diarrhœas, which had preceded the epidemic of dysentery; but it utterly failed in the latter, although employed in combination with opiates. The popular practice of burnt brandy, and light doses of castor oil, was by far more successful.

"The next year, I used calomel and astringents, but with little better success, especially with children. The year after, 1836, I used the sugar of lead, with starch and laudanum, containing alterative mercurial doses, and the other adjuvants above mentioned. With this practice, I was more successful. In 1837, I again resorted to opium more freely, and found that, with patients past the age of six years, I could control the disease perfectly; under this age, sufficient doses endangered the brain. * * * * * * * * During the late prevalence of dysentery, my practice has been to give alterative doses of calomel with rhubarb and magnesia, till the stools became bilious; then to substitute Dover's powders. I used brandy as a stimulant, with starch and laudanum injections, demulcent drinks, &c., and milk diet. Opium was freely given till it checked the tenesmus, and the calomel persevered with till the gums were touched. Afterwards, I substituted small doses of tartar emetic and nitrate of potash, avoiding nausea, but keeping the patient in a profuse perspiration. With this practice I had entire success. * * * * *.

"In one case of dysentery, which had become chronic, and had returned repeatedly after apparent cures, and to which I was called in a neighbouring town, and had under treatment when I received the Examiner, containing Dr. Meig's remarks on Mr. Hope's camphor mixture, I gave the mixture with immediate and entire success."

[In the formula for Hope's mixture, p. 286, No. 18, for T. opii., gtt. xi., read gtt. xl.—We shall be glad to have the report of the case Dr. G. speaks of.—Eds.]

M. Duges, Professor of Clinical Surgery at Montpelier, has just died of typhus fever, at the age of 41 years. M. Duges was well known in this country through his work on the diseases of the uterus, written in conjunction with Madame Boivin. He was also the author of an "Elementary Treatise on Midwifery," and of several excellent articles in the "Dictionary of Medicine," and periodical journals.

